# Friendship and self-harm: a retrospective qualitative study of young adults' experiences of supporting a friend who self-harmed during adolescence

**DOI:** 10.3389/fpsyg.2023.1221661

**Published:** 2024-02-02

**Authors:** Delfina Bilello, Ellen Townsend, Matthew R. Broome, Stephanie Burnett Heyes

**Affiliations:** ^1^School of Psychology, University of Birmingham, Birmingham, United Kingdom; ^2^Institute for Mental Health, University of Birmingham, Birmingham, United Kingdom; ^3^Self-Harm Research Group, School of Psychology, University Park, The University of Nottingham, Nottingham, United Kingdom

**Keywords:** self-harm, friendships, young people, qualitative methods, interviews, retrospective, adolescence, peers

## Abstract

**Introduction:**

Self-harm amongst young people is becoming increasingly prevalent. Understanding, responding to, and supporting young people who self-harm is vital. Friends are typically the first and sometimes the only source of support sought by adolescents who self-harm. Despite their important role as confidants, friends' perspectives and experiences remain poorly understood.

**Methods:**

We conducted retrospective qualitative semi-structured interviews, prompted by an adapted version of the Card Sort Task for Self-Harm (CaTS-FF), about the experiences of nine female young adults (18-20 years old) who supported a friend who self-harmed during adolescence. Data were analyzed using thematic analysis.

**Results:**

Four themes were developed: (1) “I did not realize my friend was on the road to self-harm”: Friends' reactions to self-harm; (2) “That's what friends do”: the role of friends; (3) The impact of supporting a friend who self-harms; and (4) “They were quite formative years”: reflecting on growth through the experience.

**Discussion:**

The present findings highlight the complex experiences of young people supporting a friend who self-harms. Despite being willing to take on the role of a supporter, participants experienced a range of difficult emotions and consequences. The temporal transition running through the four themes reflects the evolving nature of participants' attitudes, knowledge, and friendships. Overall, results highlight the unmet needs of adolescents supporting young people who self-harm, as well as identifying potential pathways to “support the supporters” toward resilience.

## Introduction

Self-harm is a complex, multi-faceted phenomenon with a “*distinctly social nature*” (Copeland et al., 2019, p. 1506). It is defined as any act of self-injury or self-poisoning, irrespective of the underlying intent (National Institute for Health and Care Excellence (NICE), 2013). Self-harm typically begins and intensifies during adolescence (Gillies et al., 2018), representing a significant concern in this age bracket, with rates ranging from 10% to 20% worldwide (Muehlenkamp et al., 2012; Lim et al., 2019). Self-harm rates have been steadily increasing in recent years in the UK (McManus et al., 2019). Adolescent self-harm may contribute to further health, social, emotional, and psychological difficulties, as well as increasing the risk of suicide (Mars et al., 2014; Hawton et al., 2020). Troublingly, adolescents who self-harm seldom access mental health or support services (McManus et al., 2019). Instead, they generally rely on informal sources of support, especially friends (Gillies et al., 2018; Geulayov et al., 2022). Therefore, it is important to understand the social context of adolescent self-harm, and especially the role and experiences of friends.

Adolescence is a socially sensitive period marked by high motivation to invest in peer relationships (Berndt, 2002; Blakemore and Mills, 2014). Friendship quantity, quality, and support can exert positive, protective effects on mental wellbeing (Van Harmelen et al., 2021) as well as predict lower rates of self-harm (Kasen and Chen, 2020). Adolescents who self-harm tend to first, and sometimes only, confide in their friends for support (Michelmore and Hindley, 2012). Doing so may help manage self-harm thoughts and urges, facilitate disclosure, and encourage formal help-seeking (Armiento et al., 2014; Giletta et al., 2015). However, having a friend who self-harms is itself a risk factor for self-harm (Jarvi et al., 2013). Friends' self-harm and depression can increase adolescents' emotional distress and likelihood of self-harming (Giletta et al., 2013; Mueller and Abrutyn, 2015). Normalization and imitation of self-harm may occur within adolescent peer groups, which may promote bonding and reinforce group identity (Jarvi et al., 2013; You et al., 2013; Young et al., 2014). As such, evidence indicates that adolescents can impact, and be impacted by, their friends' self-harm in multiple ways (Doyle, 2017). However, despite friends' distinctively important position, their experiences and outcomes remain poorly understood (Hilt et al., 2008).

Below, we briefly summarize research exploring the perspectives and experiences of friends of young people who self-harm before explaining the approach taken in the current study. First, research investigating general attitudes toward self-harm suggests adolescents typically lack knowledge and may hold stereotypical views, especially if they have no prior experience with the behavior (Doyle, 2017). Second, research exploring responses to self-harm disclosure shows that friends' prior experience can influence their reactions to disclosure, help-provision, and emotional responses (Law et al., 2009; Lloyd et al., 2018). Third, studies focusing on adolescents' experiences during the process of supporting a friend who self-harms highlight willingness and obstacles to providing help, as well as negative feelings (e.g., sadness, anger, shock) and alterations in friendship dynamics (Fisher et al., 2017; Shepherd, 2020; Hall and Melia, 2022). However, to date, research has considered these time points separately, i.e. before, during, or after disclosure. Considering these different time points on a continuum within one study may afford a comprehensive and integrated view of friends' evolving experiences over time. For instance, the changes in their attitudes, role, and friendship, as well as the consequences of supporting a friend who self-harms.

The current study adopted an explicitly temporal, retrospective approach. Doing so allows consideration of the evolving time-course of a complex life event (Banyard et al., 2010; Roach et al., 2021). Previous retrospective research on adolescents' experiences of bereavement by peer suicide highlights the impact and persistence of strong, negative feelings, as well as evidence that over time, individuals may come to accept the loss by supporting one another (Brent et al., 1993; Melhem et al., 2004; Bartik et al., 2013; Labestre and Gayoles, 2021). Equivalent research relating to self-harm is scarce. Nonetheless, these important findings highlight the potential for distress and the need for support amongst adolescent friends, reinforcing the need to better understand their experiences. To retrospectively explore friends' experiences, we used an adapted version of the Card Sort Task for Self-Harm (CaTS; Townsend et al., 2016). The CaTS is a task consisting of a pack of cards—representing thoughts, feelings, events, and behaviors—which the participant selects from and sorts along a timeline to describe their own experiences of self-harm. The use of this task provided evidence of self-harm changes over time (Townsend et al., 2016). It is possible that friends' experiences co-evolve with those of the young person who self-harms. Therefore, in the present study, the CaTS for Friends (CaTS-FF) was used for participants to describe their perceptions of their friends' self-harm. The process of card-sorting guides participants to reconstruct the causal sequence preceding and following their friend's self-harm, as well as providing a visual aid to prompt and discuss difficult topics in a scaffolded manner (Lockwood et al., 2017).

### The present study

The present study took a qualitative approach to understand the experiences of young adults who supported a friend who self-harmed during adolescence, with a focus on the social aspect of such experiences. The use of retrospective semi-structured interviews, scaffolded through the card sort task for self-harm for friends (CaTS-FF), enabled us to consider the breadth of friends' experiences over time in order to:

(a) Describe how participants perceived, experienced, and responded to their friends' self-harm.
(b) Explore how friends influenced and were influenced by the self-harm experience, including its impact on the friendship over time.
(c) Better understand the needs of adolescent supporters.

## Methods

### Sample and recruitment

The present sample consisted of nine female psychology undergraduate students (aged 18–20; *M* = 18.78, SD = 0.83) from a UK university. All except for one participant supported a female friend. Participants were recruited through a university-based research participation platform in exchange for course credit as part of a larger study that took place between October 2021 and December 2021. For the larger study, recruitment material stated that participants were potentially eligible to take part if they identified as having self-harmed, had a friend(s) who self-harmed, or both, during adolescence (age 14–17) and had access to a laptop/phone for the interview. For the present study, only individuals who identified and preferred to talk about their experiences as friends were included. Three participants reported or suggested also having experiences of self-harm (thoughts or behaviors), but were included in the present study as they preferred to talk about their experiences as friends. Ethical approval was obtained from the University of Birmingham's Science, Technology, Engineering and Mathematics Ethics Review Committee (ERN_19-1815).

### Procedure

Participants were invited to an introductory session via Zoom with the first author to receive information about the purpose and conduct of the study. Through this, they were able to familiarize themselves with the researcher and the sensitive content of the interviews and to determine whether they considered themselves eligible and comfortable to participate. Participants completed the consent form and baseline questions on Qualtrics. Consenting participants were given access to the CaTS-FF and instructions for completion. In a final online session via Zoom with the first author, participants gave verbal consent and completed the CaTS-FF, which was then used as a prompt in the semi-structured interview. Interviews were audio-recorded using secure electronic and physical audio-recording devices. At the end of the interview, participants were debriefed and signposted to appropriate sources of support.

A follow-up email was sent 2 weeks after the interview to prompt participants to share any thoughts or comments. Participants who responded to the email mainly emphasized the benefits of participation (Dazzi et al., 2014). The comments were not included in the data analysis.

### Study materials

*Baseline questionnaire:* Participants completed a demographic questionnaire (including age and gender identity) and a baseline screening question that asked which experience they preferred to primarily talk about in the interview (i.e. their own or a friend's self-harm).

**Figure 1:**
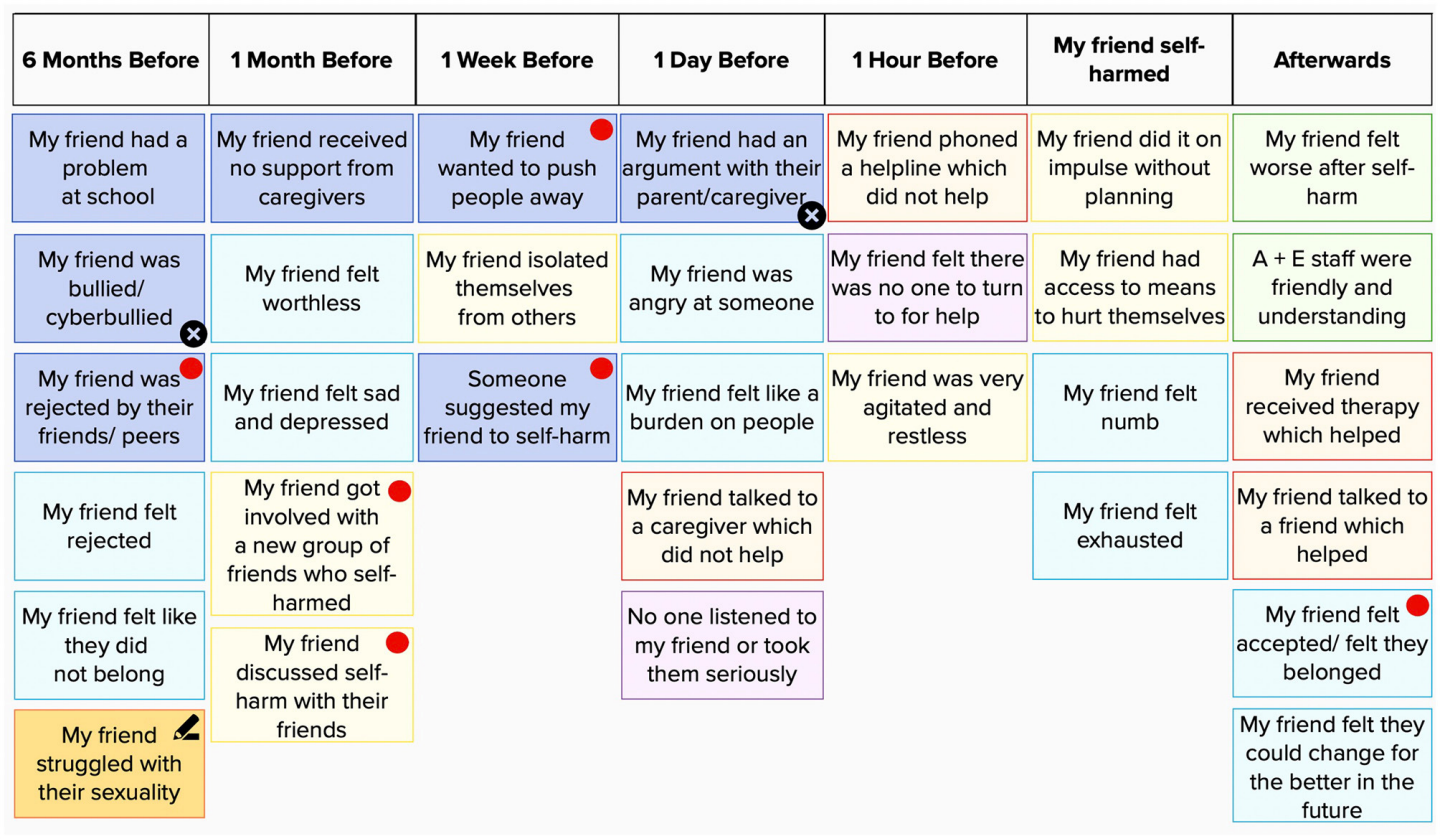
Sample CaTS-FF task on Mural. Card color denotes the category of an event or experience, e.g., thoughts, feelings, events, behaviors, and support/services. Blank cards are represented with a pen symbol. Crosses specify experiences the participant chooses not to talk about. Red dots denote examples of novel social cards developed by the research team for the present study and added to the original CaTS.

*Card sort task for self-harm for friends (CaTS-FF):* The original CaTS task was adjusted to represent participants' perceptions of their friends' self-harm experiences and surrounding psychosocial and contextual elements. Additionally, given the focus on social factors, 18 new social cards were added based on previous literature and team discussions (Heath, Ross, Toste, Charlebois and Nedecheva, 2009; Edmondson, Brennan and House, 2016; marked with a red dot in Figure 1). Furthermore, 19 original cards were simplified or combined, reducing the total number of cards to 116. Participants were given access to the CaTS-FF through a link to Mural, an online visual collaborative space, or received a paper copy by post, depending on their preference. Participants were instructed to familiarize themselves with and pick relevant cards, which were then placed along the timeline. Participants could place a sticker on cards they did not wish to discuss and could write their own cards for experiences that were not represented (see Figure 1).

*Semi-structured interview:* The participant's arranged CaTS-FF was used as a prompt to guide discussion during interviews. The semi-structured interview schedule included questions on the following topics, in line with the research aims: (a) knowledge and perceptions of self-harm and of their friend's self-harm experience; (b) participants' own experience of supporting their friend; (c) characteristics and evolution of the friendship; and (d) participants' own support needs.

*Visual analog scale (VAS):* Participants verbally rated their mood before and after the interview on a three-point VAS (bad, neutral, good). Information from the VAS was not collected; instead, it was used by the researcher to identify and act on potential distress, as in previous studies (Townsend et al., 2016).

### Epistemological position

The overarching philosophical framework driving the present research is pragmatism. This framework, placed at the intersection between realism and idealism, accepts that our understanding of the world is subjective whilst acknowledging that it can often be shared across individuals, forming relatively stable patterns of reality (Morgan, 2014). Pragmatism uses a variety of quantitative and qualitative approaches similar to the present study, which combines an objective task, the CaTS-FF, and subjective, semi-structured interviews. This was consistent with the flexibility and data-driven nature of the chosen analysis method, thematic analysis, which enables identification of common contextual factors across participants, whilst also exploring how friends subjectively interpret and make sense of their own individual experiences (Braun and Clarke, 2019).

The first author, a female researcher undertaking a psychology doctorate research degree, undertook the interview and data analysis processes. The second author, a psychology professor with expertise in the area of self-harm, guided and assisted during the data analysis.

### Data analysis

Data were transcribed verbatim by the first author. Nine transcripts, in which young people identified as friends or primarily focused on their experience as friends, were included in the analysis. Data were analyzed using thematic analysis in NVivo v12 (QSR International Pty Ltd. Version 12 2018) following Braun and Clarke's guidelines (2019). This approach analyses and considers the breadth of subjective experiences whilst addressing concrete research questions.

The first author familiarized themselves with the data by reading and re-reading all the transcripts, which were then coded inductively line-by-line. Simple descriptive codes were applied to the transcripts and were later classified based on the content they were mainly referring to (e.g., general understanding of self-harm; friendships; own experiences). At this stage, the analysis shifted and focused primarily on the social aspects of the experience—consistent with the research aims. Interpretative codes that reflected more abstract patterns in the data were developed alongside initial themes. Subsequently, discussions within the research team and consideration of the relevant literature were undertaken to contextualize, refine, and make sense of the findings. Furthermore, a reflective diary of observations made throughout both the interviews and analysis informed the aforementioned processes as well as served to reflect on the impact of the researcher during interviews, analysis, and interpretation. Altogether, through these steps and a weekly peer-review process conducted with ET, the main ideas were defined and organized into final themes, presented herein.

## Results

**Figure 2:**
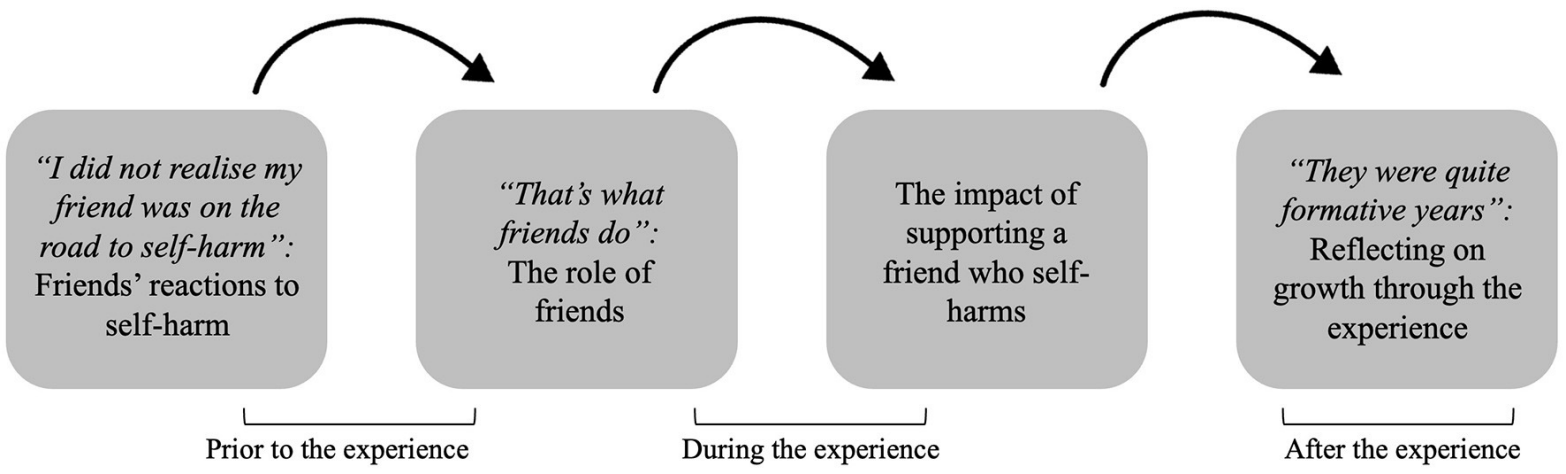
Supporting a friend who self-harms in adolescence: thematic map and temporal transitions between themes.

Following the interviews, four themes were developed: (1) “*‘I did not realize my friend was on the road to self-harm”: Friends' reactions to self-harm'* which reflect friends' initial understanding of self-harm and reaction to their friend's disclosure; (2) “*‘That's what friends do”: The role of friends'* encompasses changes in the role of friends and friendship dynamics in response to and throughout the experience; (3) ‘*The impact of supporting a friend who self-harms*' considers the challenges and negative impact of the supporter role; and (4) “*‘They were quite formative years”: Reflecting on growth through the experience'* retrospectively reflects on personal growth attributed to the experience. These themes follow a transitional sequence, from initial reactions, through the experience of supporting their friend primarily during adolescence and to current reflections on the impact of these experiences (see Figure 2).

### Theme 1: “*I did not realize my friend was on the road to self-harm”*: Friends' reactions to self-harm

All friends interviewed reported having extensive knowledge of their friends' history and experiences. “*I mean, I know quite a lot of her life history because I've known her since I was about six*” *(P8)*. However, most participants had not expected self-harm to happen to their friend, nor did they consider their friend as “someone who would self-harm.” This theme reflects friends' initial reactions toward self-harm and the sense-making process.

***Not realizing friends were “on the road to self-harm”***

Participants demonstrated an awareness of their friend's negative feelings, yet they did not “*pick up*” *(P5)* on potential warning signs and did not realize the severity of their friend's situation:

“*I mean, before, we didn't really have an idea or where this came from... we knew she was sad and she thought she was, like I said, a burden and everything. She was really anxious, but we didn't know it was ever getting to this stage of self-harm.” (P1)*

This was partly related to participants' limited knowledge and lack of experience with self-harm: “*We were all quite young when this happened, so I didn't really know much about self-harm” (P12)*. Most “*never really thought … that people would want to hurt themselves*” *(P16)*, whilst others considered that their friend's problems were “*minor problems*” *(P14)* or not “*enough of a reason to do something so seriou*s” *(P23)*.

In other cases, even when friends openly suggested having considered self-harm, participants still “*didn't think anything of it*” *(P14):*

“*She kept talking about: “Oh what if I do this, what if I do that” to us like as a friend group […] but it wasn't really just mentioning it just randomly, we were talking about the topic and she just mentioned it, so we didn't really think of it as much*.” *(P12)*

These instances demonstrate that self-harm was something participants knew little about and perceived as unlikely, which led to it initially slipping under their radar.

***My friend would never do that***

Participants' previous assumptions and knowledge about self-harm were challenged upon realizing that their friends were going through it. This led participants to question their previous beliefs, attitudes, and knowledge about self-harm and who may be at risk. Initially, some participants thought their friends “*weren't like that*.” One participant recalled telling their friend “*Wait, I wouldn't expect someone like you to do it […] this is not you*” *(P12)*. Overall, this reflects how self-harm was seen as a behavior characteristic of certain people but not their friend:

“*I didn't have any idea that she would do that … like on her phone she used to have like screensavers of depressing quotes and stuff, but I used to think: “She would never do that” because she was against that for other people*.” *(P8)*

To resolve this mismatch, participants reflected upon the reasons and circumstances leading their friend to self-harm. Some suggested it was related to a sudden change in their friend's behavior, whereby they “*acted very strangely” (P23)* and they were not the same as before. For some, this meant they had to get their “*friend back” (P5)*.

A few participants suggested that their friend knowing other people who were self-harming “*made it more normal in her (their friend's) head, than it actually is” (P16)*. Therefore, without these external social influences, their friend's self-harm “*wouldn't have started” (P16)*.

“*She kind of got involved into a new group of friends that were more, like, they had self-harmed, they would regularly talk about it, so it sort of planted the seed in her head, we think… […] I don't think she would have ever considered it before.” (P1)*

Overall, this subtheme taps into participants' process of understanding their friend's self-harm. Their pre-conceptions about self-harm and its perceived external causes had implications for how they responded to it.

### Theme 2: “*That's what friends do”*: The role of friends

All participants interviewed reported having a substantial role as sources of support in their friend's self-harm experience, often more than other adults and professionals: “*talking to like us, people like our age […] she could relate to someone” (P12)*. In some cases, friends became “*the only person she (their friend) could speak to” (P8)*. Accordingly, participants reflected on changes in friendship roles and dynamics.

***Friends will always be there to help***

All participants demonstrated a positive and understanding attitude toward helping their friend, including emotional support: “*We'd give her constant hugs and just, just every time ask her if she was okay even if she's not just to make sure that she knows that she can talk to us” (P5)*, instrumental support by helping with “*all those things that she relies on” (P23)*, and informational support by “*discussing it (self-harm)” (P1)* with their friend.

Particularly, participants emphasized their wish to “*always be there*” to support their friend by being understanding, patient, and without expecting anything in return since “*it's their choice […] if they want to talk to me or not” (P23)*. Most reflected on the importance to “*give them space and time but always be there when they need you” (P12)* and to avoid pressuring or imposing expectations on their friends.

It is important to note that initially some participants were unsure how to respond to their friend's self-harm. Many reflected how their approach changed over time as they learned how to support their friend: “*I think from my side I was more sensitive about what I said, cause like one could…. I realized that something could be triggers and stuff” (P18)*.

Overall, participants felt they were an important source of support for their friend, and they were willing to take on that role and provide unconditional support.

***Felt they had to “step up” to help their friend***

The experience of supporting a friend led to changes in friendship roles, reciprocity, and dynamics. Participants described feeling they were almost counselors and “*less of a friend, and more of like, I don't want to say therapist, but you know what I mean” (P16)* or that friendship roles were altered or reversed: “*It makes me feel tables have turned, because I used to look up to her and I'm here because of her and now it's kind of like the other way around” (P23)*.

As a consequence, some participants noted that they felt they had to “*step up as a friend” (P8)*, often because “*there was no one so it was kind of like, forced to be, like me and the other people” (P16):*

“*I was just worried cause you know it looked quite bad […] I was a close friend, but she didn't have many other people, and so I was as like: “Alright I need to kind of step up and ask otherwise it's going to get really bad.” (P17)*

In the majority of cases, participants believed they were too young, inexperienced, and “*not even vaguely equipped to deal with it” (P17)*. At the same time, they believed individuals “*need professional help and you as a friend cannot do it too much, like maybe for short term” (P23)*. Whilst they wanted to notify appropriate adults to ensure their friends received appropriate support, they did not want to “*betray” (P17)* their friend's trust nor “*feel like I'm like sabotaging my friend” (P16)* as that was seen as “*an invasion of who she (their friend) is as a person” (P8)*.

Participants' sense of responsibility and duty to help their friend was highlighted, yet this was often accompanied by feelings of being unprepared and unequipped.

***Friends parting ways***

Changes in the friendship were also noted, whereby three participants mentioned parting ways with their friend. The reasons for friendships dissolving were not necessarily attributed to self-harm *per se*. For instance, in one case it was just the product of “*drifting away” (P12)*. Instead, for other participants, it was the product of their own circumstances and mental health concerns, or a conflict with their friend that “*drove a massive breach into our (their) friendship” (P8)*.

“*It was like a hard thing you know … cause she was basically my go-to person and I had to force myself not to text her, not to tell her things […] We didn't just part ways because of the self-harm but because of what was going on in my life and how she was, you know, reacting to it.” (P23)*

Interestingly, these three participants also mentioned having considered or enacted self-harm. Whether these two aspects were related was not addressed at the time of the interview.

Overall, these instances point at the limitations of a friend's role and potential negative emotional consequences, which in some cases led participants to step away from the friendship.

### Theme 3: The impact of supporting a friend who self-harms

Throughout the interviews, friends reflected on how the experience impacted them and their own wellbeing and how, despite needing support, they seldom received it.

***“It was quite hard to deal with”***

Participants described various negative emotional consequences throughout the experience. For instance, upon learning about their friends' self-harm, participants' immediate emotional reaction was characterized by shock and “*a sense of shame” (P8)* and regret for not realizing sooner: “*Being his best friend, why didn't I realize?” (P14)*:

“*I kind of feel a lot of regret about the situation because […] If only I had known about the whole social rejection and how like angry and upset she was about all of it, it would have been a lot better.” (P17)*

Several participants suggested feeling “*a bit guilty” (P1)* as they “*should have just supported her (their friend) more” (P1)*. For others, guilt stemmed from the conflict between thinking they could support their friends more, but also wanting things to go back to normal or for their friend to “*fix themselves quickly” (P16)*. As participant 23 highlighted, “*There's always this selfish and supportive part just like fighting for it” (P23)*.

Other participants expressed feeling “*angry […] like caring angry” (P12)*. In such cases, this was often directed at the situation rather than at their friend: “*I couldn't quite control like my rage because like seeing your friend going through that … it's not anger at her but anger at the situation that she's experiencing” (P8)*.

Throughout the experience of supporting their friends, feelings shifted to being tired, drained, and exhausted: “*I think particularly the few days after I was quite drained and tired […] At that age it's quite young and quite hard to deal with” (P1)*. These feelings often stemmed from not knowing how to respond and support their friends: “*It was like quite a big burden to have. […] No matter what you did you kind of felt like what you were doing was wrong … it was like, it was very like draining to do” (P16). Feeling “quite anxious” (P8)* and uncertain of what could happen to their friend was also a recurrent thought amongst participants. Most suggested being “*more conscious that it could get there quickly” (P1)*, “*so worried that she (their friend) would do something to herself” (P8)* and scared that “*next time it would be … more severe” (P5)*.

Overall, it was apparent that friends were impacted emotionally by the self-harm, their responsibility toward their friend, and concern about the consequences it could have for their friend.

***“I needed to talk about some of it with someone”***

Most participants observed that, at the time, it did not occur to them to take particular steps to deal with the aforementioned negative feelings they experienced. Generally, they “*just like accepted it” (P18)* or they didn't think “*there was any strategies to cope, I think I was just crying it all out because there was nothing else that I could do” (P14)*.

The majority suggested feeling able to cope with the experience on their own, yet this made them feel alone and isolated. For instance, participant 8 recounted that “*it was just a matter of processing it by myself” (P8)*, yet she still found it quite hard “*to process that alone because I would've left and spoken to a friend about it, kind of like get their opinions about what I should do” (P8)*. Some participants suggested that being offered support in dealing with the situation could have been helpful:

“*I think maybe support being offered to them (to friends of people who self-harm) as well, because I think I would have benefited from that a lot if the teacher who I said, had been like: “Oh are you okay with like everything that happened?.” Like obviously, I was fine, but I think I needed to talk about some of it with someone.” (P17)*

Instead, others were able to rely on their support network, especially their friendship group, where they “*would remind each other: ‘It's not like your fault.' It was kind of, a little support thing” (P16)*. The importance of having an informal support system as well as the lack thereof was noted:

“*I think I just tried to cope with it by talking to my friends and my mum probably. […] And I think it made it less draining because we all had each other to cope with it together. I think if I was the only person who knew or outside the family, I think it would be a lot worse, because I couldn't talk to anyone or like I'm not meant to tell anyone.” (P5)*

Overall, participants struggled during the experience, yet the support sought and received varied from person to person. Participants consistently highlighted that support was valued and needed, whether they received it or not.

### Theme 4: “*They were quite formative years”*: Reflecting on growth through the experience

In retrospect, participants considered that the experience offered opportunities for personal growth and, in some cases, strengthened their friendships.

***Learning about self-harm and mental health***

Participants reflected on how having seen a close friend go through self-harm helped them acquire and develop an awareness and understanding of self-harm: “*I discovered more about the thought processes behind it and that, and like what it actually is to the person who is doing it, rather than just seeing it as someone unnecessarily harming themselve” (P16)*. One participant reflected that “*when you've seen it, it's a bit clearer” (P1)*, which mirrors how many participants became more “*aware of the signs” (P8)* and “*on the lookout for things like that” (P17)*.

Many expressed that the experience improved their awareness of mental health in general and the fact that “*mental health issues are like, are a lot more common than you think and it can just like happen, it doesn't even necessarily need a trigger” (P16)*. As one participant pointed out, the experience “*sort of opened my eyes up to like how people feel” (P14)*.

Overall, for the majority of participants, their role in their friend's self-harm experience represented “*quite formative years because you do learn that … everyone, everyone has stuff going on” (P17)*. In some cases, these also seemed to influence their attitudes, interests, and educational choices.

***Learning about supporting others***

Given their improved understanding of mental health, most participants highlighted feeling better able to support both their friends and others. They reinforced the importance of being there for and checking in with their friends and wider social network more often because “*if they know someone is there and cares, then that's a comfort to them” (P1)*:

“*I think that if you ask someone if they're okay and they say “yeah” just ask a second time, just cause like maybe that second time they see that you do really care and they would actually come out and be like: no, I'm actually not okay.” (P14)*

In turn, they also learned to be more honest with their friends and to mutually rely on one another:

“*Just be yourself and if you're having a bad day you can tell them that you're having a bad day to them as well, because then they won't feel like they're the only one in the world that has a bad day, but also they can help you as well. And it's like a good trade-off.” (P1)*

Some but not all participants described becoming “*closer” (P17)* with their friend: “*I think we'll always be good friends because of that happening […] We tried to help as much as we can and I think because of that, I think our relationship is probably stronger than it was, maybe before” (P5)*. This also seemed to expand to the larger friendship group, especially for one participant who was eventually affected by bereavement: “*It hit our friendship group really hard because he was that friend that always made us laugh and like, and that did bring us together” (P14)*.

Overall, the experience was formative and, in some cases, “*just made everyone appreciate life a lot more and like appreciate friends” (P14)*. The impact and value of friendships were highlighted: “*Sometimes like friendship groups and who you hang out with and involve with can greatly affect like … how you feel about mental health” (P12)*.

***Learning about themselves***

Finally, participants suggested becoming more aware that they themselves could also face similar issues as their friends. For several participants, the experience made them reflect on their own circumstances:

“*The positive thing I think was about myself […] just me being in a better place, you know, that made me realize that I could have been way worse, let's say that. And that made me like improve my mental health even better.” (P23)*

Consequently, the importance of taking care of their own mental health and wellbeing and “*as well as making sure other people are okay, making sure that I'm okay” (P14)* was highlighted:

“*Just remembering to take care of myself as well and remembering what could happen, obviously it's extreme, you know … if you don't take care of yourself, it might just, you might end up spiraling.” (P5)*

As a result, some suggested the experience “*made them stronger” (P5)* as they were able to develop and recognize the coping strategies and resources available to them: “*It's good to look back and realize that you do actually have strategies. […] I can deal with this, and I have these support pillars, and this is what I can do” (P1)*.

This theme highlights how, looking back on the experience, participants reflected on its formative aspects, opportunities for growth, and long-lasting impact.

## Discussion

The present study aimed to gain an in-depth understanding of the experiences of young people who supported a friend who self-harmed during adolescence through a retrospective lens. Psychology undergraduate students from a university within the UK were interviewed in this study, with discussions scaffolded using an objective tool, the CaTS-FF. Participants' subjective perceptions, thoughts, and reflections were thematically analyzed. Four main themes were developed: (a) “*I did not realize my friend was on the road to self-harm”*: friends' reactions to self-harm; (b) “That's what friends do: the role of friends”; (c) The impact of supporting a friend who self-harms; and (d) “*They were quite formative years”*: reflecting on growth through the experience. Each theme alludes to a specific stage throughout the experience, reflecting the evolution from participants' initial understanding of self-harm through the strategies and roles adopted to support their friends to the impact of the experience.

Research suggests that young people who self-harm typically consider friends the primary and sometimes the only source of support they feel able to confide in (Gillies et al., 2018; Geulayov et al., 2022). This view was shared by the friends in our sample, providing corroborating first-person evidence. Adolescent friendships have particular characteristics, such as trust, intimacy, and self-disclosure (Bagwell and Schmidt, 2013), which may be relevant in the context of self-harm.

The present results reflect that despite participants' awareness of their friend's distress and difficult circumstances, they had not anticipated self-harm. This is further demonstrated by their reactions to disclosure, including shock, surprise, and disbelief. Coinciding with studies in adults, participants reported that the self-harm was unexpected and stemmed from a change in their friends, who were “not themselves” (Sweeney et al., 2015). Interestingly, many participants reflected retrospectively on instances in which apparent disclosure occurred but was not recognized. It remains unclear whether this was the result of friends' wishes not to overreact (Knightsmith, 2018), barriers to acknowledging stigmatized behavior, or misinterpretation as a casual joke or product of the context (Sweeney et al., 2015). It is also possible that friends initially lacked sufficient knowledge of self-harm to recognize it (Doyle, 2017). Understanding why some disclosures are recognized when others may inadvertently be missed warrants further investigation to promote early intervention.

In the present study, whilst participants reported initially lacking knowledge about self-harm, common prejudices and stereotyped views about self-harm (e.g., as attention-seeking) remained in the minority (Doyle, 2017). Instead, friends often attributed the self-harm to factors outside of their friend's control, such as peer influence. Public perception studies suggest that perceived motivations for self-harm and attributions of causality may influence individuals' attitudes and helping behavior (Law et al., 2009; Nielsen and Townsend, 2018). Specifically, intrapersonal motivations and external attributions for self-harm, such as it being related to mental health struggles and uncontrollable causes, lead to more helping responses from others. Instead, interpersonal motivations for self-harm, such as being attention-seeking, and internal attributions of causality lead to less pity, more anger, and less willingness to provide support.

Altogether, this may account for friends' sympathetic and understanding attitudes and the unconditional wish to provide support observed in our study. This was a key aspect all participants reflected upon, highlighting the strength of friendship ties during this period of life. Support came in the form of checking in with their friends and actively listening to their concerns, whilst also respecting their need for privacy and allowing them time and space to share when they felt comfortable. This approach is considered positively by young people who self-harm, highlighting concrete directions for effective support (Fortune et al., 2008; Wadman et al., 2018). It is worth noting that this approach appeared to develop over time, whereby friends' initial strong reactions ultimately transitioned to an understanding standpoint focused on learning how to support their friend (Park et al., 2021), alongside an awareness that their role has limitations (Fisher et al., 2017). This transition may underlie the resolution of the initial conflict between participants' needs (to “get their friend back”; for their friend to cease self-harming) and the needs of their self-harming friend (Fisher et al., 2017). However, these findings stand in stark contrast with the perceived decrease in support over time noted by individuals who self-harm (Hasking et al., 2015). Future studies, considering the perspectives of both supportive friends and young people who self-harm, could shed light on the apparent mismatch between these two experiential narratives.

The aforementioned mismatch in perceived experiences might be related to the demands, costs, and negative consequences associated with the supporter role (Shepherd, 2020). Plausibly, the closeness and intimacy typical of adolescent friendships led to feelings of duty and responsibility toward their friend (Shepherd, 2020; Hall and Melia, 2022). Yet, most participants mentioned feeling “like” therapists or counselors, alluding to a sense of being unequipped and unprepared for the role. Even though friends recognized the importance of seeking appropriate adult support, they did not want to betray their friend's trust by doing so (Fisher et al., 2017; Roach et al., 2021). The need to find new ways to “support the supporters” is an important conclusion from the current study.

Inner conflict and feelings of constant worry, anxiety, and guilt were mentioned across all interviews. These are common experiences observed amongst friends (Bresin et al., 2013), families (Oldershaw et al., 2008), and professionals alike (Boukouvalas et al., 2019). Such feelings may potentially be heightened in the former group, whose emotion regulation strategies are still developing in tandem with decreased reliance on parents vs. peers for support (Zimmer-Gembeck and Skinner, 2010; Reindl et al., 2016). Therefore, coping with giving support may become increasingly difficult for adolescent friends in the context of self-harm. Notably, lack of access to adult support, for their friends and for themselves, was reported by participants. In turn, friends may face difficulties talking about their own issues with their friend who is self-harming to avoid burdening them. This may leave friends feeling overwhelmed and unsupported. The wish to receive support for their own experiences supporting a self-harming adolescent friend is particularly noted in the current study (cf. Shepherd, 2020).

It is important to note that in this specific sample, more extreme manifestations of distress, such as self-harm “contagion,” were not often mentioned. Arguably, this may be partly attributed to self-selection bias operating at several levels, including participants with both first-person and supportive-friend experiences of self-harm electing to talk about the former. However, three specific participants stood out in the present study given that they suggested also having considered or engaged in self-harm and also described having parted ways with their friend. This “parting of ways” warrants further investigation as a potential strategy individuals may employ to limit personal impact from the experience and reduce the demands of their role (Fisher et al., 2017).

Despite the significant challenges and negative impact experienced at the time, the majority of individuals sampled reflected on some degree of personal growth through the experience. Particularly, participants noted improved mental health literacy and understanding of self-harm, which in most cases increased confidence in supporting others. For some, the experience strengthened relationships with close friends and the wider friendship group. This mirrors the posttraumatic growth observed amongst some survivors of bereavement by peer suicide (Labestre and Gayoles, 2021). This, however, appeared to be contingent upon the outcomes experienced by their friend. For instance, the majority suggested their friends had recovered or were “in a better place.” This may not be the case for every young person; hence, pathways toward risk and resilience should be explored further.

Finally, it is important to highlight the utility of the CaTS as a tool facilitating exploration of how participants' experiences evolved over time. Notably, the CaTS is a non-threatening, accessible tool and shared visual prompt for discussing this sensitive topic, alleviating the potential emotional impact of verbalizing certain experiences (Townsend et al., 2016). In the future, the CaTS could be used as a visual tool to promote perspective-taking amongst pairs of friends and individuals who self-harm, enabling direct mutual comparison of their perspectives and experiences regarding the same event (Shepherd, 2020). The flexibility and adaptability of the tool present important applications for both research and practice.

### Limitations

These findings need to be interpreted, taking into account the sample studied. Given that this was a self-selected sample of psychology students, it is plausible that participants had high levels of awareness and felt comfortable discussing sensitive topics. In turn, it is plausible that participants whose experiences had been resolved in some way were more willing to take part. Qualitative research focuses on the in-depth understanding of a particular topic amongst a specific subgroup, rather than generalizing findings (Polit and Beck, 2010). Nonetheless, this highlights potential barriers to engaging individuals with a range of experiences, including those who preferred to maintain their friend's privacy, who did not consider themselves a close friend, or who experienced predominantly negative outcomes (Bartik et al., 2013). The experiences of self-harm across all adolescent supporters, irrespective of position, closeness, and experience, should be investigated further.

In turn, the sample is fairly homogeneous and limited in terms of diversity. For example, it consisted exclusively of young people who identified as female, the majority of whom supported a female friend. This aligns with evidence that females present higher rates of self-harm and of having friends who also self-harm (Copeland et al., 2019). It is not known whether these findings also represent experiences amongst other gender identities. This identifies an important evidence gap given that these groups may present unique friendship dynamics and barriers to help-seeking and help-provision alike (Oransky and Marecek, 2009; McDermott et al., 2018).

### Conclusion and recommendations

Extensive literature has highlighted the importance of adolescent friends as primary confidants and supporters of young people who self-harm. However, little research has focused on their unique experiences and the potential long-term consequences associated with this role.

The present findings align with evidence that young people who self-harm rely on and confide in their friends for support, and we found that friends were willing to take on the role of supporter. Despite initial strong, negative emotional reactions, friends' attitudes toward self-harm evolved into an understanding approach focused on supporting their friend. In most cases, this led to changes in individuals' attitudes toward self-harm and mental health. However, it is important to note how negative feelings prevailed throughout the experience, still impacting the individual and their friendship in the present. In particular, the current study highlights an apparent mismatch between the support needs of adolescent supporters and the support they received.

Altogether, these findings highlight the need to provide support to young people, irrespective of their apparent circumstances. This could be undertaken via universal strategies aimed at enhancing young people's mental health literacy and self-harm/suicide awareness (e.g., SEYLE study, Wasserman et al., 2015). In turn, targeted intervention strategies should be developed and specifically tailored to the friends of young people who self-harm. We suggest these include training in communication skills, boundaries, and acknowledging their own emotional needs and potential limitations of their role. This should be accompanied by training for professionals and families to facilitate understanding of the needs of adolescent supporters. Overall, considering the perspectives of young people supporting a friend who self-harms should be at the forefront of research to improve our understanding of young people's experiences in the context of self-harm and to develop better interventions both incorporating and targeting friends.

## Data availability statement

The datasets presented in this article are not readily available because the data generated during and/or analyzed during the current study are not publicly available nor are they available on request as consent for research data sharing was not obtained from participants. Requests to access the datasets should be directed to DXB984@bham.ac.uk.

## Ethics statement

The studies involving humans were approved by University of Birmingham's Science, Technology, Engineering and Mathematics Ethics Review Committee (ERN_19-1815). The studies were conducted in accordance with the local legislation and institutional requirements. Written informed consent for participation was obtained from participants.

## Author contributions

DB: conceptualization, methodology, formal analysis, investigation, writing–original draft, and visualization. SBH and ET: conceptualization, methodology, validation, resources, writing—review and editing, supervision, and funding. MB: conceptualization, resources, writing—review and editing, supervision, and funding. All authors contributed to the article and approved the submitted version.
